# Loss in Later Life: Partnership and Health After the Death of an Adult Child

**DOI:** 10.1093/geroni/igaf122.253

**Published:** 2025-12-31

**Authors:** Rose Anderson, Christine Mair

**Affiliations:** University of Maryland, Baltimore County, Baltimore, Maryland, United States; University of Maryland, Baltimore County, Baltimore, Maryland, United States

## Abstract

The death of a child is one of life’s most devastating experiences. The resulting health implications can extend into parents’ later years, yet research on how child loss is associated with older adults’ physical and mental well-being remains limited. Specifically, there is a need for additional studies on the extent to which partnership buffers or exacerbates these patterns. Using data from the 2018 Health and Retirement Study (HRS) dataset (N = 15,432), we analyze how child loss is associated with psychological and physical health outcomes among partnered and unpartnered older adults. Contrary to initial hypotheses, partnership did not buffer associations between child loss and lower psychological and physical health. Furthermore, unpartnered and “kinless” (unmarried and childless) older adults who experienced child loss faced similar declines in health as their partnered counterparts. The results of this analysis suggest that the nature of child loss, the different grieving contexts between partnered and unpartnered individuals, and the need for broader emotional and social support systems may explain this pattern. These findings highlight the importance of developing grief support approaches that address heterogeneity among bereaved parents. Future studies should investigate the potential for a wide array of social relationships and their potential buffering role following the loss of an adult child.

